# Microparticles from red blood cell transfusion products induce a strong inflammatory host response

**DOI:** 10.1186/cc14416

**Published:** 2015-03-16

**Authors:** M Straat, M Van Hezel, A Boing, R Nieuwland, R Van Bruggen, N Juffermans

**Affiliations:** 1 Academic Medical Center, Amsterdam, the Netherlands; 2 Sanquin Blood Cell Research, Amsterdam, the Netherlands

## Introduction

Red blood cell (RBC) transfusion is associated with increased morbidity and mortality in the critically ill. Adverse effects of transfusion may be mediated by red blood cell storage lesion. In this study, we hypothesized that MPs from stored RBC bags would induce a more pronounced host response than MPs from fresh RBC bags and that this response is dose dependent.

## Methods

MPs were isolated by high-speed centrifugation from red blood cell transfusion bags stored for 2 to 7 (fresh) or 25 to 35 (stored) days. Whole blood from healthy volunteers was incubated with supernatant from the bags either containing MPs or depleted from MPs (*n *= 12 bags per group). Controls were incubated with PBS as a negative and LPS (10 ng/ml) as a positive control. Cytokines in supernatant were measured by ELISA. Data are expressed as medians and interquartile ranges.

## Results

Supernatant from blood bags containing MPs strongly induced production of all cytokines compared with supernatant without MPs, a reaction which equaled that of LPS stimulation. MPs from stored RBC bags induced higher production of TNF (868 (263 to 1,625) vs. 2,596 (407 to 3,040) pg/ml, *P *= 0.049), IL-6 (1,088 (234 to 3,716) vs. 6,952 (1,507 to 21,990), *P *= 0.042) and IL-8 (1,333 (535 to 3,569) vs. 5,562 (833 to 13,904), *P *= 0.081) compared with MPs from fresh RBC bags. There was no difference in IL-10 responses between groups (8.0 (3.9 to 32.1) vs. 3.9 (3.9 to 22.2), *P *= 0.390). The host response was dose dependent both for fresh and stored MPs. In addition, the same amount of older MPs induced a stronger host response compared with fresh MPs.

## Conclusion

MPs from RBC transfusion bags induce a strong proinflammatory response, which is largely negated when MPs are removed. This MP-mediated response depends both on the amount of MPs as well as on alterations in MPs as a result of storage.

